# Anthroponotic Enteric Parasites in Monkeys in Public Park, China

**DOI:** 10.3201/eid1810.120653

**Published:** 2012-10

**Authors:** Jianbin Ye, Lihua Xiao, Jingbo Ma, Meijin Guo, Lili Liu, Yaoyu Feng

**Affiliations:** East China University of Science and Technology, Shanghai, People’s Republic of China (J. Ye, J. Ma, M. Guo, L. Liu, Y. Feng);; and Centers for Disease Control and Prevention, Atlanta, Georgia, USA (J. Ye, L. Xiao)

**Keywords:** Cryptosporidium, Giardia, Enterocytozoon bieneusi, rhesus monkey, baboon, zoonosis, anthroponotic, protozoa, parasites, enteric

## Abstract

*Cryptosporidium* spp., *Giardia duodenalis*, and *Enterocytozoon bieneusi* were detected in 45, 35, and 116 of 411 free-range rhesus monkeys, respectively, in a popular public park in the People’s Republic of China. Most genotypes and subtypes detected were anthroponotic, indicating these animals might be reservoirs for human cryptosporidiosis, giardiasis, and microsporidiosis.

## Abstract 2

Some infections are known to spread from animals to humans; others, from humans to animals. And some are not so neatly categorized. Recently, 3 diarrhea-causing parasites of humans were found in apparently healthy monkeys in a public park in China. How the monkeys became infected is unknown. It is possible that the parasites were spread from humans. No matter how the monkeys became infected, park visitors are at risk for infection from the monkeys. Park visitors, who are allowed to feed and play with the monkeys, should be informed that they can get diarrhea directly from the monkeys or from contaminated lake or drinking water.

Cryptosporidiosis, giardiasis, and microsporidiosis are enteric diseases in humans and are mainly caused by *Cryptosporidium* spp., *Giardia duodenalis*, and *Enterocytozoon bieneusi*, respectively (*1*–*3*). These protozoan parasites are also commonly found in animals and are considered zoonotic. However, the role of nonhuman primates in the transmission of the diseases remains unclear because few studies have been conducted on the genetic characteristics of the parasites in these animals. In a recent study in Kenya, 5 (2.0%) and 13 (5.5%) of 235 captive baboons had human-pathogenic *C. hominis* subtypes and *E. bieneusi* genotypes, respectively. This finding implies that nonhuman primates might be reservoirs for human cryptosporidiosis and microsporidiosis (*4*). We determined the genotypes and subtypes of *Cryptosporidium* spp., *G. duodenalis*, and *E. bieneusi* in free-range rhesus monkeys in a popular public park to assess the potential for transmission of these parasites from rhesus monkeys to humans.

## The Study

In November 2010, we collected 411 fecal specimens from rhesus monkeys (*Macaca mulatta*) in Qianling Park, Guiyang, People’s Republic of China (www.qlpark.cn). The park, a major tourist attraction of the city, is visited by 10,000–70,000 persons each day. It has the highest number (≈700) of domesticated free-range monkeys in China, which originated from a troop of 20 animals in 1985. Visitors are allowed to bring or buy food to feed the animals, watch them from a short distance, or play with them (Figure, panel A).

**Figure:**
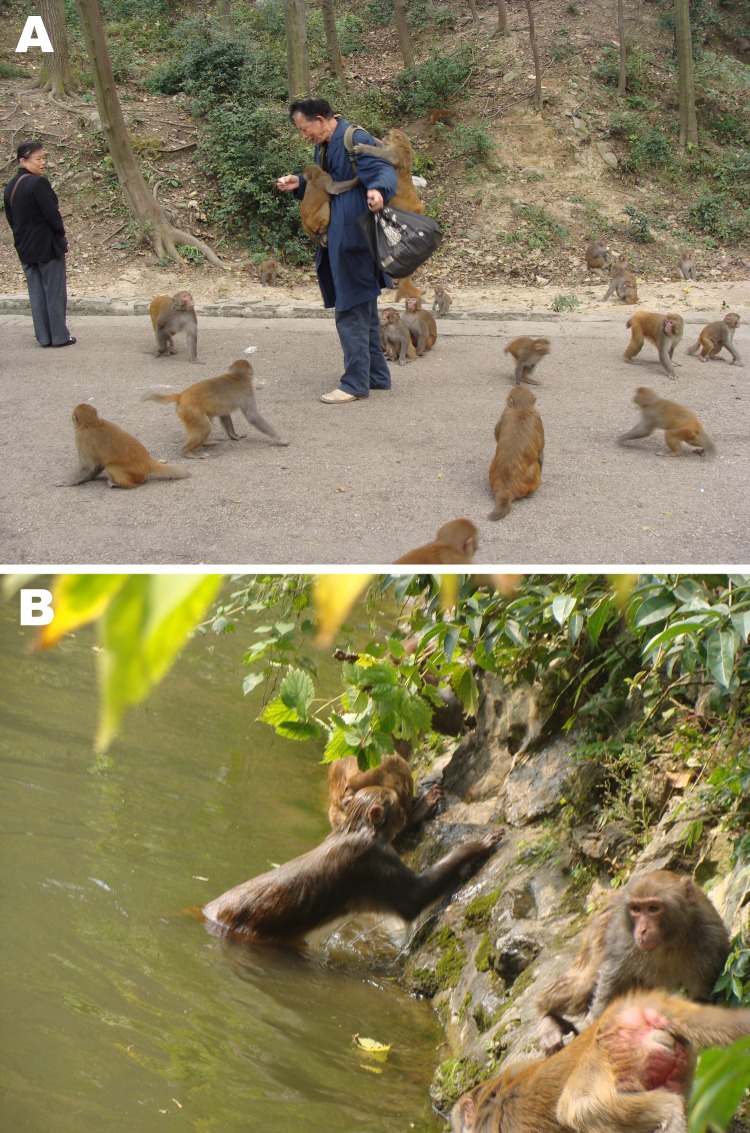
Potential zoonotic and waterborne pathways of parasites in Qianling Park, Guiyang, China. A) Close contact of rhesus monkeys with humans. B) Potential contamination of recreational water with pathogens from rhesus monkeys.

We collected fecal droppings at 3 locations with different animal densities. A total of 187 specimens were collected from the Macaque Garden, where animal density was the highest, at ≈400 animals in a small open area between 2 mountains. Another 74 specimens were collected from the Tanquan Spring area, where animal density was the lowest. The remaining 150 specimens were collected from the Hongfu Temple, which had moderate animal density. Twenty-three 100-mL grab samples of high-turbidity water were collected at various points of a small lake near the Macaque Garden and Tanquan Spring, where the rhesus monkeys frequently bathed (Figure).

We detected *Cryptosporidium* spp., *G. duodenalis* subtypes, and *E. bieneusi* genotypes in the fecal specimens and differentiated them by using PCR and sequence analysis of the small subunit rRNA gene (*5*), triosephosphate isomerase gene (*6*), and ribosomal internal transcribed spacer (*4*), respectively. We similarly analyzed water samples after concentrating pathogens by centrifugation at 3,000 × *g* for 15 min. We subtyped *C. hominis* and *C. parvum* by using sequence analysis of the 60-kDa glycoprotein gene (*5*). We analyzed each specimen at least 2× by using PCR, with the inclusion of positive and negative controls in each run. We used the χ^2^ test to compare differences in rates of each parasite.

We detected *Cryptosporidium* spp. in 45 (10.9%) of the 411 fecal specimens, belonging to 3 species: *C. hominis* (39 specimens), *C. parvum* (5), and *C. felis* (1). The rate at Macaque Garden (16.6%) was significantly higher than at Hongfu Temple (6.0%; p = 0.003) and Tanquan Spring (6.8%; p = 0.038) (Table). Among 44 specimens successfully subtyped, 7 subtypes in 5 subtype families were identified: 4 families (Ia, Id, Ie, and If) of *C. hominis* and 1 family (IIc) of *C. parvum* (Table). The most common subtypes were IaA13R8 (8 specimens), IdA20 (13), IeA11G3T3 (13), and IIcA5G3a (5).

**Table:** Anthroponotic enteric parasites in free-range rhesus monkeys (*Macaca mulatta*) and water samples in Qianling Park, Guiyang, China*

Species, genotype, or subtype	GenBank accession no.	Fecal specimens positive for organism			Water samples positive for organism, n = 23
		Macaque Garden, n = 187	Hongfu Temple, n = 150	Tanquan Spring, n = 74	
*Cryptosporidium*					
* C. hominis*					
IaA13R7	EU095261	2	0	0	0
IaA13R8	JX000568†	6	2	0	2
IaA14R7	JX000569†	1	0	1	0
IdA20	EU095265	10	3	0	0
IeA11G3T3	DQ665689	8	3	2	7
IfA16G2	JX000570†	1	0	0	0
Unknown‡	NA	0	0	0	2
* C. parvum*					
IIcA5G3a	AY738195	2	1	2	0
* C. felis*		1	0	0	0
Subtotal (mean %; 95% CI)§		31/187 (16.6; 10.7–22.4)	9/150 (6.0; 2.1–9.9)	5/74 (6.8; 0.8–12.7)	11/23 (47.8; 19.6–76.1)
*Giardia duodenalis*					
Assemblage A2	U57897	10	0	0	0
Assemblage B					
B1	AY368164	3	7	1	8
B2d	JX000562†	0	2	0	0
B3d	JX000563†	0	1	0	0
B4d	JX000564†	2	0	0	0
B5d	JX000565†	2	3	0	3
B6d	JX000566†	2	0	0	0
B7d	JX000567†	0	1	0	0
Unknown‡	NA	0	1	0	1
Subtotal (mean %; 95% CI)§		19/187 (10.2; 5.6–14.7)	15/150 (10.0; 4.9–15.1)	1/74 (1.4; 0–4.0)	12/23 (52.2; 22.7–81.7)
*Enterocytozoon bieneusi*					
Peru11	AY371286	46	21	2	4
WL15	AY237223	15	9	4	5
EbpC	AY371279	4	0	0	1
Type IV	AY371277	6	0	0	2
LW1d	JX000571†	0	0	0	1
Macaque1¶	JX000572†	0	0	1	0
Macaque2¶	JX00057†	1	0	0	0
Unknown‡	NA	4	2	1	0
Subtotal (mean %; 95% CI)§		76/187 (40.6; 31.5–49.8)	32/150 (21.3; 13.9–28.7)	8/74 (10.8; 3.3–18.3)	13/23 (56.5; 25.8–87.2)

*Values are number of positive samples unless otherwise indicated. NA, not applicable. †From this study. ‡PCR positive but sequence unavailable. §No. positive /no. tested (% positive; 95% CI). ¶New subtypes or genotypes identified during this study.

*G. duodenalis* was identified in 35 (8.5%) of the 411 fecal specimens. The rates at Macaque Garden (10.2%; p = 0.016) and Hongfu Temple (10.0%; p = 0.018) were significantly higher than at Tanquan Spring (1.4%). All positive specimens except for 1 were successfully genotyped and subtyped and belonged to assemblages A (10) and B (24). All assemblage A isolates belonged to subtype A2 (10 specimens). In assemblage B, 7 subtypes were identified: 1 known subtype in 11 specimens and 6 new subtypes at low frequencies (Table).

*E. bieneusi* was identified in 116 (28.2%) of the 411 fecal specimens. The occurrence rate at Macaque Garden (40.6%) was significantly higher than at Hongfu Temple (21.3%; p = 0.00016) and Tanquan Spring (10.8%; p = 0.000033). Among the 109 specimens successfully sequenced, 6 genotypes were identified: 4 known genotypes (Peru11 [69 specimens], WL15 [28], EbpC [4], and Type IV [6]) and 2 new genotypes at low frequencies (Table).

*Cryptosporidium* spp., *G. duodenalis*, and *E. bieneusi* were detected in 11 (47.8%), 12 (52.2%), and 13 (56.5%), respectively, of the 23 water samples collected from the lake where the animals bathed. Fewer *C. hominis* and *G. duodenalis* subtypes and *E. bieneusi* genotypes were detected in water samples than in fecal specimens. Most of the common *C. hominis* (IaA13R8 and IeA11G3T3) and *G. duodenalis* (B1 and B5) subtypes and all common *E. bieneusi* genotypes (Peru11, W15, EbpC, and Type IV) in animals were found in water samples (Table). We deposited unique nucleotide sequences obtained in GenBank under accession nos. JX000562–JX000573.

## Conclusions

All *C.* *hominis* and *C. parvum* subtypes found in this study are well-known parasites of humans and have rarely been found in animals. The C. *hominis* subtype families Ia, Id, Ie, and If had been reported in humans and urban wastewater in China (*5*,*7*–*9*). Although it has not been found in humans in China, the *C. parvum* IIc subtype family identified in rhesus monkeys in this study is a well-known anthroponotic parasite in developing countries (*1*).

The *G. duodenalis* subtypes found in Qianling Park are also major pathogens in humans. The subtype A2 of assemblage A is a common pathogen in humans in most areas studied and is less frequently found in animals than the A1 subtype (*2*). The dominant B1 subtype found in Qianling Park is also identical to an assemblage B subtype (GenBank accession no. GU564280) previously identified in humans in China (*9*).

Most *E. bieneusi* genotypes identified in this study had also been reported in humans. Among the dominant *E. bieneusi* genotypes, Peru11 had been seen only in humans and baboons (*3*,*4*). Although genotypes IV, EbpC, and WL15 have been reported in animals, they are common parasites of humans in many areas (*3*).

The origin of *Cryptosporidium* spp., *G. duodenalis*, and *E. bieneusi* parasites in the rhesus monkey population is not clear. Because these parasites are common human pathogens, they could have been introduced by humans. However, rhesus monkeys can be natural hosts of these organisms, as supported by recent identification of some of these organisms in newly captive baboons from rural and forested areas (*4*). Regardless of the initial origin of the parasites, they can be transmitted efficiently among rhesus monkeys, as supported by the higher occurrence of *Cryptosporidium* spp., *G. duodenalis*, and *E. bieneusi* at places with higher animal density.

Our results indicate that rhesus monkeys in close contact with humans are commonly infected with human-pathogenic *C. hominis*, *C. parvum*, and *G. duodenalis* subtypes and *E. bieneusi* genotypes. Therefore, they can serve as reservoirs of human cryptosporidiosis, giardiasis, and microsporidiosis. Zoonotic transmission of infection from these monkeys can occur directly by close contact of monkeys and humans (Figure, panel A), or indirectly through contamination of drinking water or recreational water (Figure, panel B). Efforts should be made to educate the public about the potential risk for zoonotic transmission of enteric pathogens from rhesus monkeys and to minimize contamination of drinking and recreational water by parasites of rhesus monkey origin.
